# Rumen Fluid Metabolomics Analysis Associated with Feed Efficiency on Crossbred Steers

**DOI:** 10.1038/s41598-017-02856-0

**Published:** 2017-06-06

**Authors:** Virginia M. Artegoitia, Andrew P. Foote, Ronald M. Lewis, Harvey C. Freetly

**Affiliations:** 1 University of Nebraska-Lincoln, Department of Animal Science, Lincoln, NE 68583 USA; 2 USDA, ARS, U.S. Meat Animal Research Center, Clay Center NE, Nebraska, 68933 USA

## Abstract

The rumen has a central role in the efficiency of digestion in ruminants. To identify potential differences in rumen function that lead to differences in average daily gain (ADG), rumen fluid metabolomic analysis by LC-MS and multivariate/univariate statistical analysis were used to identify differences in rumen metabolites. Individual feed intake and body-weight was measured on 144 steers during 105 d on a high concentrate ration. Eight steers with the greatest ADG and 8 steers with the least-ADG with dry matter intake near the population average were selected. Blood and rumen fluid was collected from the 16 steers 26 d before slaughter and at slaughter, respectively. As a result of the metabolomics analysis of rumen fluid, 33 metabolites differed between the ADG groups based on t-test, fold changes and partial least square discriminant analysis. These metabolites were primarily involved in linoleic and alpha-linolenic metabolism (impact-value 1.0 and 0.75, respectively; *P* < 0.05); both pathways were down-regulated in the greatest-ADG compared with least-ADG group. Ruminal biohydrogenation might be associated with the overall animal production. The fatty acids were quantified in rumen and plasma using targeted MS to validate and evaluate the simple combination of metabolites that effectively predict ADG.

## Introduction

Improving production efficiency of cattle by increasing meat produced per amount of feed offered would result in economic and environmental benefits^1^. However, the physiological mechanisms and the shifts in the metabolic pathways that result in increased efficiency are not fully elucidated. Digestion and nutrient absorption are considered important sources of variation in cattle growth efficiency^2^. Changes in ruminal microbial communities have been associated with differences in feed efficiency of beef cattle^3, 4^. Hence, differences in feed efficiency might be related to changes in the metabolism in the cattle rumen.

Metabolomics, has been a useful approach to characterize the metabolism of rumen fluid in dairy cows^5–8^. For instance, phenotype differences on residual feed intake were associated with specific ruminal microbes and targeted metabolic pathway in dairy cows^8^. By definition when feed efficiency is defined as residual feed intake there is a difference in feed intake between the groups. The differences in feed intake can greatly influence the microbial communities. We have chosen an alternative model that classifies steers based on divergent body weight gain at similar feed intakes in order to avoid changes associated with feed intake. We hypothesized that cattle that differed in ADG had differences in rumen metabolism. Therefore, untargeted metabolomics profile based on UPLC-quadrupole time of flight tandem mass spectrometry (qTOF-MS/MS) coupled to univariate and multivariate analysis was used to identify rumen metabolites that differed with feed efficiency and determine biomarkers in rumen fluid and plasma for ADG.

## Results

### Characteristics of steers in the two groups

The DMI did not differ between ADG groups (10.10 ± 0.05 kg/d; *P* = 0.41); however, ADG was greater (*P* < 0.01) in the greatest ADG group (1.96 ± 0.02 kg/d) than the least ADG group (1.57 ± 0.02 kg/d). The feed:gain ratio was 5.15 and 6.43 in greatest-ADG and the least-ADG, respectively.

### Data quality assessment of metabolomics and fatty acids quantification

The non-targeted UPLC-qTOF-MS/MS metabolite profiling of the ruminal fluid was carried out to differentiate least-ADG vs. greatest-ADG steers. For the repeatability, the relative standard deviation (RSD) for peak intensity ranged from 1.26% to 5.37%, and retention time from 0.05% to 0.25% in the QC sample. For the precision, the RSD for peak intensity ranged from 2.5% to 8.1% and for retention time ranged from 0.03% to 0.31% in the discovery samples. Fatty acids were quantified in ruminal fluid and plasma by UPLC-q-TOF-MS/MS isotope dilution. Linearity was achieved for all fatty acids (r^2^ > 0.98). The limit of detection (LOD) of this analysis was <0.1 ng/ml. For the repeatability, the RSD for concentration was <2%, and retention time was <0.12% in the LOD samples. Recovery ranged between 87% to 112%.

### Rumen metabolomics profiling

**Figure 1:**
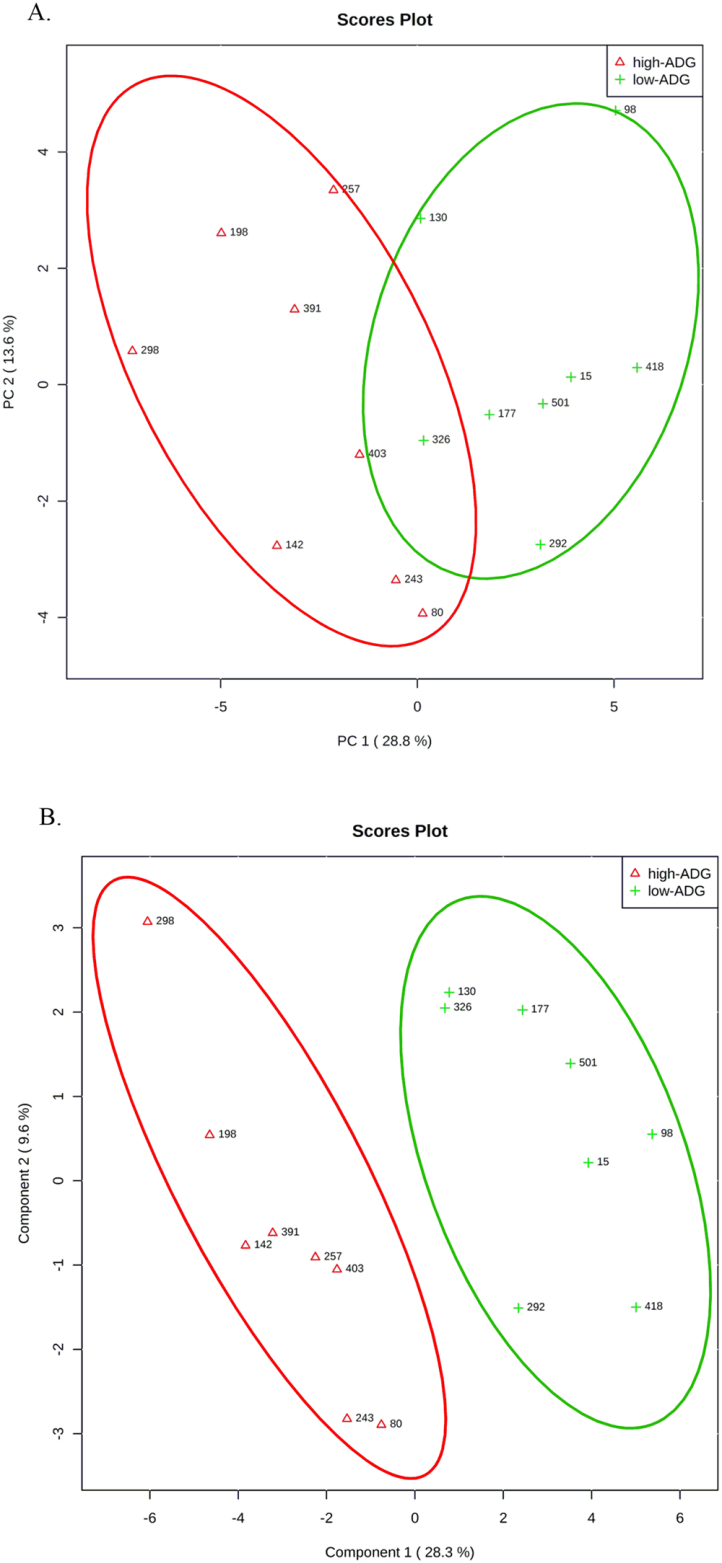
Ruminal metabolomic profile of the steers with the greatest average daily gain (high-ADG; open red triangle) and the least ADG (low-ADG; green plus) with similar average dry matter intake. (**A**) Principal component analysis for UPLC-qTOF 90 metabolites identified to differ between ADG by t-test (*P* < 0.1). (**B**) Partial Least Square-Discriminant Analysis for UPLC-qTOF 90 metabolites identified to differ between ADG by t-test (*P* < 0.1). One data point represents one steer.

**Figure 2:**
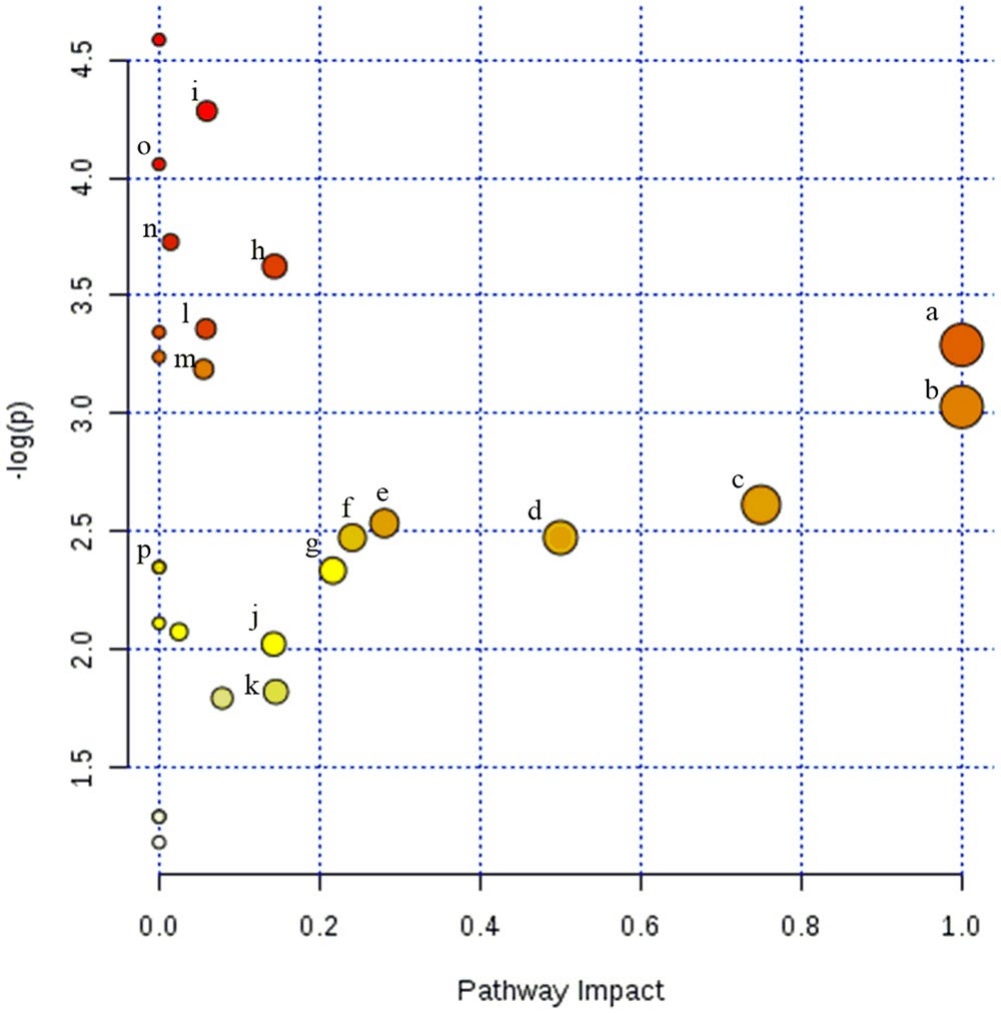
Ruminal metabolomics pathway analysis by MetaboAnalyst 3.0 Software on the steers with the greatest average daily gain compare to the least ADG with similar average dry matter intake according to *Bos taurus* KEGG pathway database. UPLC-qTOF 90 metabolites identified to differ between ADG by t-test (*P* < 0.1). (a) Alpha-linolenic acid metabolism, (b) linoleic metabolism (c) taurine and hypotaurine metabolism d) phenylalanine, tyrosine and tryptophan (e) sphingolipids (f) phenylalanine (g) retinol (h) histidine (i) primary bile biosynthesis (j) glycerophospholipid (k) tyrosin (l) porphyrin and chlorophyll (m) steroid hormone biosynthesis (n) glutathione (o) Riboflavin (p) Gluconeogenesis. The darker the color and larger the size represent higher *P-*value from enrichment analysis and greater impact from the pathway topology analysis, respectively.

**Figure 3:**
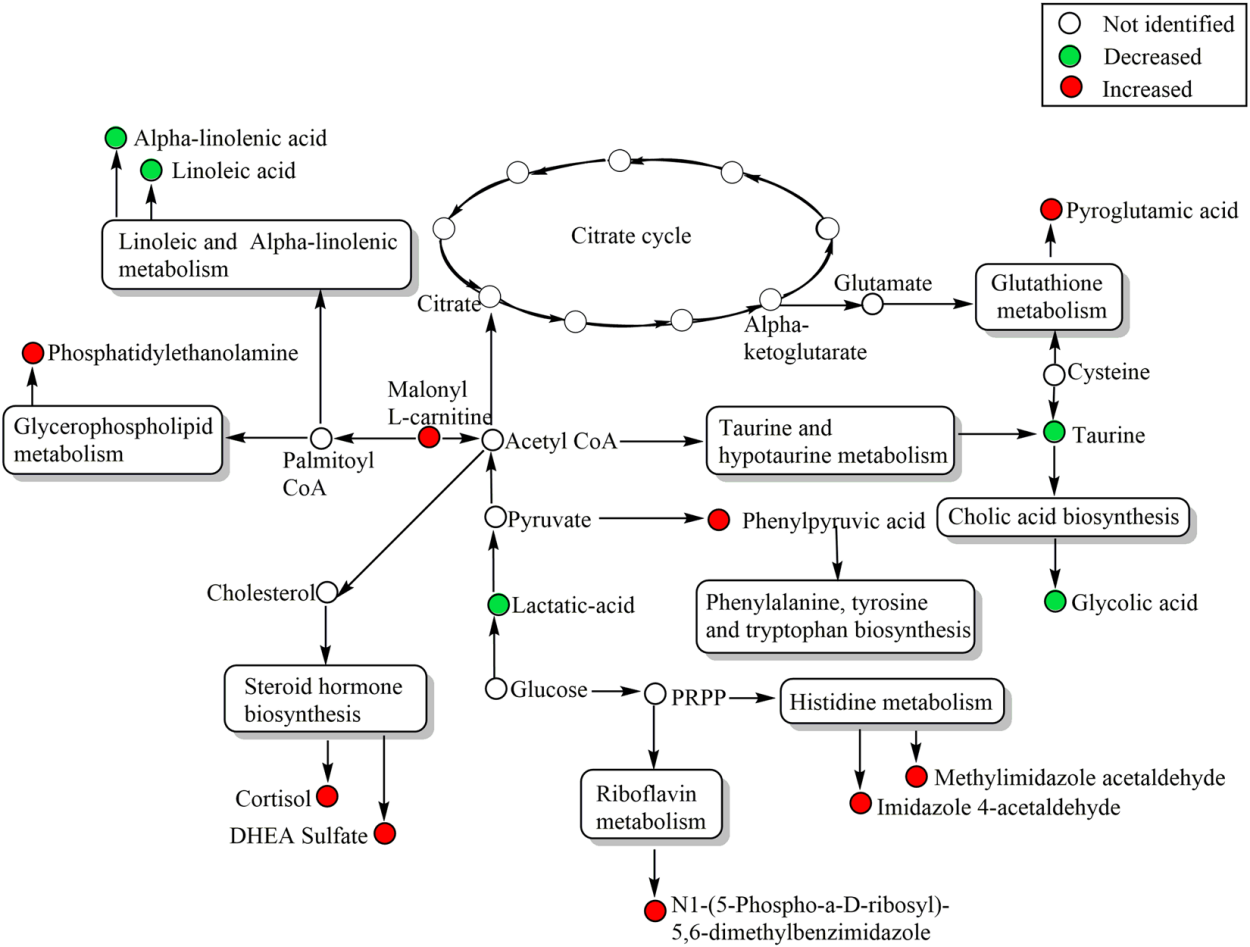
The integrative ruminal metabolic pathway changes on the steers with the greatest average daily gain compare to the least ADG with similar average dry matter intake according to *Bos taurus* KEGG pathway database. UPLC-qTOF 90 metabolites identified to differ between ADG by t-test (*P* < 0.1).

Overall, 1,429 unique metabolites (162-polar/490-non-polar metabolites positively ionized and 472-polar/305-non-polar metabolites negatively ionized) were identified. Ninety of the metabolites were identified to differ between the least and greatest ADG steers based on *t*-test (*P* < 0.1; Table 1). Using the 90 putative metabolites, principle component analysis (PCA, Fig. 1a), and pathway analysis (Figs 2 and 3, Table 2) demonstrated that the metabolome between ADG groups differed. Linoleic and alpha-linolenic acid metabolism were the two most relevant metabolic pathways down-regulated (impact value = 1 and *P* < 0.05) while phenylalanine, tyrosine and tryptophan biosynthesis was up-regulated (impact value = 0.5 and *P* < 0.08). Despite of the fact that pyruvate metabolism had no impact (only 1 hit), the level of lactic acid was 43% lower (*P* = 0.06) in the greatest-ADG steers.

**Table 1:** Identification of ruminal fluid metabolites for feed efficiency.

Metabolites	Formula	Adducts	RT (min)	Ion (m/z)	Mass error (ppm)	ID	Fold change
Alloxan	C_4_H_2_N_2_O_4_	M−H	15.87	140.99	1.97	BMDB02818	1.27
12,13-DHOME	C_18_H_34_O_4_	M−H	10.17	313.24	1.47	BMDB04705	−1.43
Kynurenic acid	C_10_H_7_NO_3_	M−H	2.19	188.03	3.25	BMDB00715	−1.96
Tauroursodeoxycholic Acid	C_26_H_45_NO_6_S	M−H	1.80	498.29	0.73	BMDB00874	−6.74
Celastrol	C_29_H_38_O_4_	M−H	12.29	449.27	2.25	BMDB02385	1.40
DG(22:0/20:3(5Z,8Z,11Z)/0:0)	C_45_H_82_O_5_	M+H	14.63	703.62	3.66	BMDB07603	2.02
Pentadecanoic acid	C_15_H_30_O_2_	M−H	13.03	241.22	1.06	BMDB00826	−1.70
Malonyl-L-carnitine	C_10_H_17_NO_6_	M−H	1.95	246.10	0.40	BMDB02095	1.30
Pterin	C_6_H_5_N_5_O	M−H	13.03	162.04	2.64	BMDB00802	1.48
N1-(5-Phospho-a-D-ribosyl)-5,6-dimethylbenzimidazole	C_14_H_19_N_2_O_7_P	M+H	8.67	359.09	2.19	BMDB03882	1.76
Pteroyl-D-glutamic acid	C_20_H_23_N_7_O_7_	M+H	10.78	474.17	1.47	BMDB02140	1.17
N-Acryloylglycine	C_5_H_7_NO_3_	M+H	12.40	130.05	4.82	BMDB01843	1.13
Eicosenoic acid^2^	C_20_H_38_O_2_	M−H	14.59	309.28	0.99	BMDB02231	−1.62
Pyroglutamic acid	C_5_H_7_NO_3_	M−H	13.28	128.03	4.73	BMDB00267	1.62
All-trans-heptaprenyl diphosphate	C_28_H_49_N_3_O_16_	M+H	10.46	655.38	3.81	BMDB12187	−1.51
5-Dodecenoic acid	C_12_H_22_O_2_	M+H	10.35	199.17	4.97	BMDB00529	−1.38
3-Oxooctadecanoic acid	C_18_H_34_O_3_	M+H	10.35	299.26	2.80	BMDB10736	−1.33
Androsterone sulfate	C_19_H_30_O_5_S	M+H	8.67	371.19	2.75	BMDB02759	1.23
DG(18:3(9Z,12Z,15Z)/15:0/0:0)	C_36_H_64_O_5_	M+H	17.45	577.48	4.31	BMDB07300	1.43
Linoleic acid	C_18_H_32_O_2_	M+H	10.35	281.25	2.59	BMDB00673	−1.21
20-Carboxyleukotriene B4	C_20_H_30_O_6_	M+H	14.26	367.21	1.21	BMDB06059	−1.14
Harderoporphyrin	C_35_H_36_N_4_O_6_	M+H	12.11	609.27	3.55	BMDB00683	−2.56
Sebacic acid	C_10_H_18_O_4_	M−H	7.31	201.11	2.59	BMDB00792	1.38
Oleamide	C_18_H_35_NO	M+H	15.07	282.28	3.66	BMDB02117	1.13
Glycocholic acid	C_26_H_43_NO_6_	M−H	5.52	464.30	0.96	BMDB00138	−1.94
Chitin	C_28_H_49_N_3_O_16_	M+H	10.57	684.32	3.56	BMDB03362	1.41
Imidazole-4-Acetaldehyde	C_5_H_6_N_2_O	M−H	1.36	109.04	3.19	BMDB03905	1.13
Vaccenic acid	C_18_H_34_O_2_	M−H	13.39	281.25	0.16	BMDB03231	−1.37
Calcitroic acid	C_23_H_34_O_4_	M+H	11.20	375.25	2.29	BMDB06472	1.13
Alpha-Linolenic acid	C_18_H_30_O_2_	M+H	9.80	279.23	1.08	BMDB01388	−2.23
12a-Hydroxy-3-							
Oxo-choladienic acid	C_24_H_34_O_4_	M−H	11.59	385.24	1.49	BMDB00385	−1.35
5-Sulfosalicylic acid	C_7_H_6_O_6_S	M+H	13.46	219.00	1.77	BMDB11725	1.51
2-Octenedioic acid	C_8_H_12_O_4_	M−H	9.52	171.07	2.13	BMDB00341	1.50
Methylimidazole acetaldehyde	C_6_H_8_N_2_O	M+H	0.47	125.071	1.14	BMDB04181	1.13
DG(16:0/20:3(5Z,8Z,11Z)/0:0)	C_39_H_70_O_5_	M+H	14.12	619.52	−0.98	BMDB07110	−1.09
Taurallocholic acid	C_26_H_45_NO_7_S	M−H	2.22	514.28	1.05	BMDB00922	−8.30
Glycoursodeoxycholic acid	C_26_H_43_NO_5_	M−H	7.39	448.30	−1.25	BMDB00708	−2.02
Taurine	C_2_H_7_NO_3_S	M−H	1.23	124.01	0.35	BMDB00251	−1.45
L-Thyronine	C_15_H_15_NO_4_	M−H	1.85	272.09	1.27	BMDB00667	1.26
7a,12a-Dihydroxy-3-oxo-4-cholenoic acid	C_24_H_36_O_5_	M+H	9.84	405.26	−4.65	BMDB00447	−1.09
Pyridinoline	C_18_H_28_N_4_O_8_	M+H	9.37	429.20	1.44	BMDB00851	−1.21
3-Methoxybenzenepropanoic acid	C_10_H_12_O_3_	M−H	1.91	179.07	1.21	BMDB11751	1.14
17-Hydroxylinolenic acid	C_18_H_30_O_3_	M+H	9.77	295.23	−0.57	BMDB11108	−1.12
Stearidonic acid	C_18_H_28_O_2_	M+H	8.63	277.22	1.79	BMDB06547	−1.11
1,25-Dihydroxyvitamin D3-26,23-lactone	C_27_H_40_O_5_	M−H	13.39	443.28	0.55	BMDB00969	1.16
3-Oxodecanoic acid	C_10_H_18_O_3_	M−H	1.88	185.12	−0.94	BMDB10724	−1.26
L-Acetylcarnitine	C_9_H_17_NO_4_	M+H	3.64	204.12	1.82	BMDB00201	−2.37
Dihydrozeatin-O-glucoside	C_16_H_25_N_5_O_6_	M+H	1.16	384.19	1.04	BMDB12214	1.35
L-Lactic acid	C_3_H_6_O_3_	M−H	0.36	89.02	−0.72	BMDB00190	−1.43
Retinoic acid	C_20_H_28_O_2_	M−H	1.03	299.20	2.24	BMDB01852	1.68
5-Hydroxytryptophol	C_10_H_11_NO_2_	M−H	1.35	176.07	0.27	BMDB01855	1.08
18-Hydroxycortisol	C_21_H_30_O_6_	M+H	13.49	379.21	4.86	BMDB00418	−1.13
Octadecanedioic acid	C_18_H_34_O_4_	M−H	10.17	313.24	−1.47	BMDB00782	−1.29
Cortisol	C_21_H_30_O_5_	M+H	12.98	363.22	−0.48	BMDB00063	1.08
MG(18:0/0:0/0:0)	C_21_H_42_O_4_	M+H	11.93	359.32	−0.30	BMDB11131	1.12
Donepezil	C_24_H_29_NO_3_	M+H	8.55	380.22	−4.55	BMDB05041	−1.11
MG(14:1(9Z)/0:0/0:0)	C_17_H_32_O_4_	M−H	13.83	299.22	−4.37	BMDB11531	1.15
L-Glutamic acid 5-phosphate	C_5_H_10_NO_7_P	M−H	6.36	226.01	−0.56	BMDB01228	1.25
Indole-5,6-quinone	C_8_H_5_NO_2_	M−H	1.12	146.02	2.63	BMDB06779	−1.29
PE(22:4(7Z,10Z,13Z,16Z)/22:5(4Z,7Z,10Z,13Z,16Z))	C_49_H_80_NO_8_P	M+H	13.46	842.57	−1.09	BMDB09603	1.10
Deoxypyridinoline	C_18_H_28_N_4_O_7_	M+H	11.01	413.20	−1.82	BMDB00569	−1.27
5-L-Glutamylglycine	C_7_H_12_N_2_O_5_	M+H	0.42	205.08	−0.56	BMDB11667	−1.54
MG(P-18:0e/0:0/0:0)	C_21_H_42_O_3_	M+H	12.73	343.32	2.10	BMDB11153	1.25
5-Hydroxy-N-formylkynurenine	C_11_H_12_N_2_O_5_	M−H	0.65	251.07	−1.15	BMDB04086	−1.19
PS(14:0/18:2(9Z,12Z))	C_38_H_70_NO_10_P	M+H	14.12	732.48	−4.02	BMDB12336	−1.13
9S-HPODE	C_18_H_32_O_4_	M−H	6.94	311.22	−1.54	BMDB06940	−1.29
Beta-Cortol	C_21_H_36_O_5_	M−H	9.37	367.25	−3.46	BMDB05821	−1.61
Arachidic acid	C_20_H_40_O_2_	M−H	14.63	311.30	−1.69	BMDB02212	−1.28
Ursodeoxycholic acid	C_24_H_40_O_4_	M−H	6.44	391.28	1.98	BMDB00946	−1.58
4alpha-Formyl-4beta-methyl-5alpha-cholesta-8,24-dien-3beta-ol	C_29_H_46_O_2_	M+H	11.26	427.36	3.55	BMDB12167	1.21
LysoPC(20:5(5Z,8Z,11Z,14Z,17Z))	C_28_H_48_NO_7_P	M+ H	10.65	542.32	−0.58	BMDB10397	−1.18
Cortol	C_21_H_36_O_5_	M−H	9.07	367.25	−3.82	BMDB03180	−1.79
(R)−3-Hydroxy-hexadecanoic acid	C_16_H_32_O_3_	M−H	11.01	271.23	−1.15	BMDB10734	−1.19
LysoPC(18:2(9Z,12Z))	C_26_H_50_NO_7_P	M+H	10.65	520.34	2.85	BMDB10386	1.42
Naringenin	C_15_H_12_O_5_	M−H	1.13	271.06	1.25	BMDB02670	1.25
p-Cresol glucuronide	C_13_H_16_O_7_	M−H	1.87	283.08	0.88	BMDB11686	1.71
Ubiquinone	C_14_H_18_O_4_	M−H	3.40	249.11	−1.39	BMDB02012	1.09
Indoleacetaldehyde	C_10_H_9_NO	M−H	1.77	158.06	2.30	BMDB01190	1.19
Hyodeoxycholic acid	C_24_H_40_O_4_	M−H	8.95	391.29	2.24	BMDB00733	−1.92
13-L-Hydroperoxylinoleic acid	C_18_H_32_O_4_	M−H	6.94	311.22	0.37	BMDB03871	−1.29
9-OxoODE	C_18_H_30_O_3_	M−H	1.96	293.21	0.87	BMDB04669	−1.30
Prostaglandin F2a	C_20_H_34_O_5_	M−H	8.15	353.23	1.24	BMDB01139	−1.40
4-Fumarylacetoacetic acid	C_8_H_8_O_6_	M−H	8.73	199.03	1.54	BMDB01268	−1.30
5-L-Glutamyl-taurine	C_7_H_14_N_2_O_6_S	M−H	1.31	253.04	2.36	BMDB04195	1.36
D-Erythrose 4-phosphate	C_4_H_9_O_7_P	M−H	11.41	199.00	1.36	BMDB01321	1.32
L-Tyrosine	C_9_H_11_NO_3_	M+H	12.58	182.08	1.95	BMDB00158	1.26
LysoPE(0:0/15:0)	C_20_H_42_NO_7_P	M−H	9.73	438.26	4.84	BMDB11502	−1.25
Phenylpyruvic acid	C_9_H_8_O_3_	M−H	1.19	163.04	2.23	BMDB00205	1.14
PIP2(16:0/16:1(9Z))	C_41_H_79_O_19_P_3_	M+H	11.23	969.46	4.95	BMDB10033	−1.22

UPLC-qTOF metabolites identified (*P* < 0.1), RT, retention time; ID, identification matched from bovine metabolome database matched, fold changes comparing high-ADG vs. low-ADG steers.

**Table 2:** Result from ruminal fluid pathway analysis.

Metabolic pathway	Total Cmpd	Hits	*P*	−log(p)	Impact
Biosynthesis of unsaturated fatty acids	42	4	0.01	4.58	0.00
Primary bile acid biosynthesis	46	2	0.01	4.28	0.06
Riboflavin metabolism	11	1	0.02	4.06	0.00
Glutathione metabolism	26	1	0.02	3.73	0.01
Histidine metabolism	14	2	0.03	3.62	0.14
Porphyrin and chlorophyll metabolism	25	1	0.03	3.36	0.06
Pentose phosphate pathway	19	1	0.04	3.35	0.00
alpha-Linolenic acid metabolism	9	2	0.04	3.29	1.00
Amino sugar and nucleotide sugar metabolism	37	1	0.04	3.24	0.00
Steroid hormone biosynthesis	67	2	0.04	3.19	0.06
Linoleic acid metabolism	5	2	0.05	3.03	1.00
Taurine and hypotaurine metabolism	7	1	0.07	2.61	0.75
Sphingolipid metabolism	21	1	0.08	2.54	0.28
Phenylalanine, tyrosine and tryptophan biosynthesis	4	2	0.08	2.47	0.50
Phenylalanine metabolism	9	2	0.08	2.47	0.24
Glycolysis or Gluconeogenesis	26	1	0.10	2.35	0.00
Pyruvate metabolism	22	1	0.10	2.35	0.00
Retinol metabolism	17	1	0.10	2.33	0.22
Arginine and proline metabolism	44	1	0.12	2.11	0.00
Glycosylphosphatidylinositol(GPI)-anchor biosynthesis	14	1	0.13	2.08	0.02
Glycerophospholipid metabolism	29	2	0.13	2.02	0.14
Tyrosine metabolism	42	2	0.16	1.82	0.15
Tryptophan metabolism	41	2	0.17	1.79	0.08
Ubiquinone and other terpenoid-quinone biosynthesis	3	1	0.27	1.29	0.00
Aminoacyl-tRNA biosynthesis	64	1	0.27	1.29	0.00
Arachidonic acid metabolism	36	1	0.31	1.18	0.00

Total Cmpd = Total is the total number of compounds in the pathway; Hits is the actually matched number from the user uploaded data; *P* is the original *P*-value calculated from the enrichment analysis; Impact is the pathway impact value calculated from pathway topology analysis.

Principal component analysis and score plot from supervised PLS-DA were conducted on the 90 putative ruminal metabolites. The analysis revealed a clear clustering between least-ADG and greatest-ADG steers (Fig. 1b), which suggest that ruminal biochemistry changed according to ADG classification. The *P*-value for 100 permutation was *P* = 0.04, indicating that the PLS-DA model was valid (PLS-DA One component: accuracy = 0.81, R^2^ = 0.77, Q^2^ = 0.59).

Thirty-three of the 90 metabolites differed between the groups (P < 0.05) after adjusting the *p*-value for false discovery rate (Table 3). The 33 potential metabolites were characterized according to* t*-test value, VIP, and AUC (Table 3). These 33 metabolites were confirmed using the MS/MS spectra by chemically intelligent peak matching algorithm from the Mass-Fragment application manager software. Eighteen metabolites, such as alpha-linolenic acid and vaccenic acid, decreased in the greatest-ADG group, whereas 16 metabolites, such as pteroyl-d-glutamic, alloxan, malonyl-carnitine, and oleamide increased in the greatest-ADG steers. The VIP values were the result of separated metabolites from PLS-DA score plot. In total, 17 of the 33 metabolites had VIP-value >1 (*P* < 0.05). The metabolites with the highest VIP-value (>1.7) were tauroursodeoxycholic acid, kynurenic acid, and glycolic acid, which were associated with lower intensity in the greatest-ADG vs. least-ADG steers.

**Table 3:** Identification of candidate biomarkers for feed efficiency on ruminal fluid.

Identified metabolite	Formula	Adducts	RT (min)	Ion (m/z)	Mass error (ppm)	VIP	Fold change	AUC	*P*	FDR
Alloxan	C_4_H_2_N_2_O_4_	M−H	15.87	140.99	1.97	1.01	1.27	0.89	<0.01	0.047
12,13-DHOME	C_18_H_34_O_4_	M−H	10.17	313.24	1.47	0.92	−1.43	0.89	<0.01	0.047
Kynurenic acid	C_10_H_7_NO_3_	M−H	2.19	188.03	3.25	1.86	−1.96	0.89	0.01	0.047
Tauroursodeoxycholic acid	C_26_H_45_NO_6_S	M−H	1.80	498.29	0.73	2.51	−6.74	0.84	0.01	0.047
Celastrol	C_29_H_38_O_4_	M−H	12.29	449.27	2.25	0.71	1.40	0.89	0.01	0.047
DG(22:0/20:3(5Z,8Z,11Z)/0:0)	C_45_H_82_O_5_	M + H	14.63	703.62	3.66	1.57	2.02	0.86	0.01	0.047
Pentadecanoic acid^2^	C_15_H_30_O_2_	M−H	13.03	241.22	1.06	1.26	−1.70	0.86	0.01	0.047
Malonyl-L-carnitine	C_10_H_17_NO_6_	M−H	1.95	246.10	0.40	1.05	1.30	0.86	0.01	0.047
Pterin	C_6_H_5_N_5_O	M−H	13.03	162.04	2.64	1.24	1.48	0.84	0.02	0.047
N1-(5-Phospho-a-D-ribosyl)-5,6-dimethylbenzimidazole	C_14_H_19_N_2_O_7_P	M + H	8.67	359.09	2.19	0.67	1.76	0.86	0.02	0.047
Pteroyl-D-glutamic acid	C_20_H_23_N_7_O_7_	M + H	10.78	474.17	1.47	0.70	1.17	0.83	0.02	0.047
N-Acryloylglycine	C_5_H_7_NO_3_	M + H	12.40	130.05	4.82	0.67	1.13	0.84	0.02	0.047
Eicosenoic acid^2^	C_20_H_38_O_2_	M−H	14.59	309.28	0.99	1.00	−1.62	0.81	0.02	0.047
Pyroglutamic acid	C_5_H_7_NO_3_	M−H	13.28	128.03	4.73	1.43	1.62	0.86	0.02	0.047
All-trans-heptaprenyl diphosphate	C_28_H_49_N_3_O_16_	M + H	10.46	655.38	3.81	1.31	−1.51	0.83	0.03	0.047
5-Dodecenoic acid	C_12_H_22_O_2_	M + H	10.35	199.17	4.97	0.97	−1.38	0.80	0.03	0.047
3-Oxooctadecanoic acid	C_18_H_34_O_3_	M + H	10.35	299.26	2.80	0.82	−1.33	0.81	0.03	0.047
Androsterone sulfate	C_19_H_30_O_5_S	M + H	8.67	371.19	2.75	0.86	1.23	0.83	0.03	0.047
DG(18:3(9Z,12Z,15Z)/15:0/0:0)	C_36_H_64_O_5_	M + H	17.45	577.48	4.31	1.04	1.43	0.88	0.03	0.047
Linoleic acid^*^	C_18_H_32_O_2_	M + H	10.35	281.25	2.59	0.97	−1.21	0.81	0.03	0.047
20-Carboxyleukotriene B4	C_20_H_30_O_6_	M + H	14.26	367.21	1.21	0.75	−1.14	0.75	0.03	0.047
Harderoporphyrin	C_35_H_36_N_4_O_6_	M + H	12.11	609.27	3.55	1.71	−2.56	0.78	0.03	0.047
Sebacic acid	C_10_H_18_O_4_	M−H	7.31	201.11	2.59	0.74	1.38	0.80	0.03	0.047
Oleamide	C_18_H_35_NO	M + H	15.07	282.28	3.66	0.64	1.13	0.83	0.04	0.047
Glycocholic acid	C_26_H_43_NO_6_	M−H	5.52	464.30	0.96	1.86	−1.94	0.78	0.04	0.047
Chitin	C_28_H_49_N_3_O_16_	M + H	10.57	684.32	3.56	0.63	1.41	0.80	0.04	0.047
Imidazole-4-acetaldehyde	C_5_H_6_N_2_O	M−H	1.36	109.04	3.19	0.62	1.13	0.79	0.04	0.047
Vaccenic acid	C_18_H_34_O_2_	M−H	13.39	281.25	0.16	1.01	−1.37	0.80	0.04	0.047
Calcitroic acid	C_23_H_34_O_4_	M + H	11.20	375.25	2.29	0.56	1.13	0.80	0.04	0.047
Alpha-Linolenic acid^2^	C_18_H_30_O_2_	M + H	9.80	279.23	1.08	1.09	−2.23	0.80	0.04	0.047
12a-Hydroxy-3-oxo-choladienic acid	C_24_H_34_O_4_	M−H	11.59	385.24	1.49	1.26	−1.35	0.83	0.04	0.047
5-Sulfosalicylic acid	C_7_H_6_O_6_S	M + H	13.46	219.00	1.77	1.14	1.51	0.75	0.05	0.049
3-Octenedioic acid	C_8_H_12_O_4_	M−H	9.52	171.07	2.13	1.05	1.50	0.77	0.05	0.049

RT, retention time; VIP, value of importance in projection; Fold changes comparing the greatest average daily gain steers (*n* = 8) with the least average daily gain steers (*n* = 8) on ruminal fluid metabolites; AUC, area under the curve; *P*, treatment effect result from t-test; FDR, false discovery rate *p*-adjustment.

^2^Metabolites were quantified by liquid chromatogram tandem mass spectrometry using isotope dilution.

### ROC analysis and quantification of fatty acids

Based on the findings of the non-targeted metabolomic analysis fatty acids were quantified in rumen fluid (Table 4) and plasma (Table 5). The performance of the ruminal metabolites as biomarkers was assessed using ROC curves. According to the accepted classification of biomarker utility, candidate markers of AUC greater or equal to 0.9 are considered “excellent”, which was not the case for any of the 33 most discriminating markers (Table 3). However, discrimination between the greatest- and least-ADG groups were classified as “good” (0.8 to 0.9) for 31 of the biomarkers and “fair” (0.7 to 0.8) for 2 of the biomarkers.

**Table 4:** Fatty acid concentrations in ruminal fluid on steers with the least and greatest average daily gain.

Fatty acids (µg/mL)	Group^1^		SEM	AUC^2^	*P*	FDR^3^
	Least-ADG	Greatest-ADG				
Pentadecanoic acid (C15:0)	1.94	1.41	0.04	0.91	<0.01	0.01
Palmitic acid (C16:0)	245.5	224.1	6.65	0.70	0.13	0.17
Linoleic acid (C18:2)	28.07	19.19	2.56	0.75	0.10	0.14
Alpha-Linolenic acid (C18:3)	0.023	0.015	0.002	0.85	0.02	0.08
Eicosanoic acid (C20:0)	15.51	13.34	1.06	0.77	0.16	0.19

^1^Groups were least-ADG steers (n = 8), with the least average daily gain and greatest-ADG steers (*n* = 8), with the greatest average daily gain, with groups having similar dry matter intake. ^2^AUC = area under the curve calculated by receiver-operator characteristic curve analysis. ^3^FDR = False discovery rate *p*-adjustment.

**Table 5:** Fatty acid concentrations in plasma on steers with the least and greatest average daily gain.

Fatty acids (µg/mL)	Group^1^		SEM	AUC^2^	*P*	FDR^3^
	Least-ADG	Greatest-ADG				
Pentadecanoic acid (C15:0)	0.575	0.573	0.394	0.53	0.53	0.53
Palmitic acid (C16:0)	11.34	10.95	1.007	0.62	0.78	0.78
Stearic acid (C18:0)	10.21	8.230	0.575	0.80	0.03	0.08
Linoleic acid (C18:2)	2.511	2.112	0.386	0.56	0.48	0.48
Alpha-linolenic acid (C18:3)	0.037	0.032	0.002	0.73	0.09	0.14
Eicosanoic acid (C20:0)	0.117	0.209	0.058	0.62	0.82	0.82
Arachidonic acid (C20:4)	0.251	0.170	0.085	0.84	0.06	0.10
Docosahexaenoic acid (C22:6n3)	0.025	0.020	0.002	0.65	0.20	0.24

^1^Groups were least-ADG steers (*n* = 8), with the least average daily gain, and greatest-ADG steers (*n* = 8), with the greatest average daily gain, with groups having similar dry matter intake. ^2^AUC = area under the curve calculated by receiver-operator characteristic curve analysis. ^3^FDR = false discovery rate *p*-adjustment.

**Figure 4:**
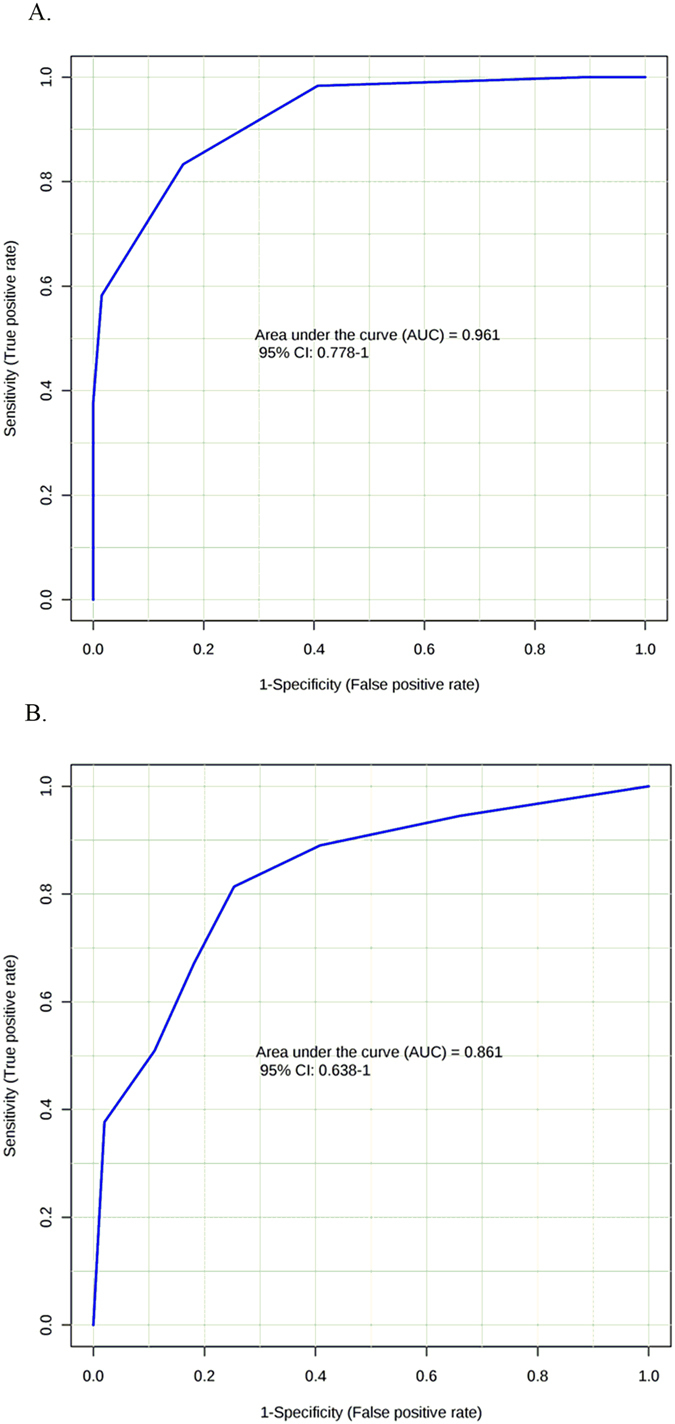
Receiver operating curves (ROC) of fatty acids for the differential identification of feed efficiency on steers. **(A**) Rumen fluid ROC using the combination of pentadecanoic acid, palmitic acid, linoleic acid and alpha-linolenic acid. (**B**) Plasma ROC using the combination of arachidonic acid/docosahexanoic acid ratio and alpha-linolenic acid.

The ruminal fatty acids concentration of pentadecanoic, linoleic, and alpha-linolenic acids were lower in greatest-ADG than least-ADG steers (*P* ≤ 0.1, Table 4). As a result of the ROC analysis of the ruminal fatty acids, the AUC values of pentadecanoic acid were the highest among single ruminal biomarkers. However, the ROC curve performance combining ruminal concentration of pentadecanoic acid, palmitic, linoleic and alpha-linolenic acid using PLS-DA model was AUC = 0.961 (95% C.I. of 0.78 to 1.0) with a 87.5% sensitivity and specificity (Fig. 4a).

Plasma fatty acids were tested as a potential biomarker to classify ADG groups. Plasma concentration of pentadecanoic acid, linoleic acid, eicosanoic acid and plamitic acid did not differ between ADG group (Table 5). However, plasma concentration of stearic acid, arachidonic acid, and alpha-linolenic acid were greater in the least-ADG vs. greatest-ADG steers (*P* < 0.10; Table 5). The AUCs of the ROC for single plasma biomakers are shown in Table 5. Only the combination of plasma alpha-linoleic acid and arachidonic/docosahexanoic acid ratio presented a slightly greater AUC value (AUC = 0.861; 95% confidence interval: 0.638 to 1 with 87.5% sensitivity and 62.5% specificity; Fig. 4b) than the AUC of arachidonic acid in plasma (AUC = 0.84; Table 5).

## Discussion

There is a need to understand the underlying physiological mechanisms that contribute to variation in feed efficiency. The production traits of beef cattle have been improved through animal selection using traditional quantitative genetics approaches^9^; however, large phenotypic variation remains unexplained. In this study we analyzed rumen fluid which is a mixture of compounds originating from the diet, animal, and microbs. Using this experimental model where diet and intake have been fixed group differences are most likely associated with difference in animal physiology or microbial metabolism.

In our study, metabolic profiling of rumen fluid based on the UPLC-qTOF-MS/MS metabolomics analysis coupled with univariate and multivariate statistical methods, was used to provide a comprehensive approach to identify metabolites that differentiate steers with greatest-ADG from least-ADG.

The composition of bovine ruminal fluid is characterized by phospholipids, inorganic ions, gases, amino acids, dicarboxylic acids, fatty acids, volatile fatty acids, glycerides, carbohydrate and cholesterol esters; many are microbial fermentation products that occur under the anaerobic condition found in the rumen^10^. Pathway analysis typically uses a subset of the data that has met a threshold level rather than the entire data set^11–13^. In our study we chose to use the 90 metabolites that were different based on the t-test between the groups rather than the more restrictive 33 metabolites that were identified after adjusting the p-value for false discovery rate. Linoleic and alpha-linolenic metabolic pathways were the most significant impact from the analysis of ruminal fluid metabolic pathway. Based on the identification of fatty acid metabolism differing between the ADG group in the non-targeted analysis a targeted analysis of fatty acids was conducted and supported the original findings. Based on ROC analysis only ruminal pentadecanoic acid had high sensitivity and specificity to predict ADG in steers. To improve the prediction, we compared the combination of fatty acids in the ruminal fluid, and the combination of pentadecanoic acid, palmitic acid, linoleic and alpha-linolenic acid could, collectively, more accurately discriminate least- and greatest-ADG steers, with higher sensitivity and specificity that the separate biomarkers. Ruminal long-chain fatty acid concentrations are indicative of active lipolysis, biohydrogenation and microbial fatty acid synthesis in the rumen^14^. Ruminants fed a finishing ration receive linoleic acid in large quantity and less alpha-linolenic acid, mainly as triglycerides^15^. Before these unsaturated fatty acids can be rapidly hydrogenated by microbes to more saturated end products, lipases, galactosidases and phospholipases produced by ruminal microbes release the nonesterified fatty acids from the ration^14^. Biohydrogenation of linoleic and alpha-linolenic acid both have an initial isomerization step producing conjugated *cis-9,trans-11* acid, which then undergoes hydrogenation of its *cis* double bonds resulting in *trans-11-*ocatdecanoic acid (trans-vaccenic acid, 11-eladic acid), with lastly hydrogenation to stearic acid^15^. This activity represents several biochemical pathways depending on the microbial ecosystem which yields a variety of intermediate fatty acids in different concentrations^16^. Particularly odd-chain fatty acid, such as pentadecanoic acid are exclusively produced in relatively high levels by rumen microbial fermentation and microbial *de-novo* lipogenesis^17^.

Previous studies of the rumen microbiome from steers found that changes in the abundances of microbial population were associated with feed efficiency group^3, 4, 18^. However, *in vivo* microbial communities composition associated with changes in biohydrogenation have had low correlation^19, 20^ and limited explanation of it association^21^. Therefore, further integration of omics technology might be a useful strategy to understand how the shift in the ruminal bacteria population affect the ruminal metabolism.

As a result of changes in fatty acid profile in ruminal fluid, we sought to quantify the fatty acids in plasma to determine potential candidates for biomarkers for feed efficiency, and their associated levels in ruminal fluid. Plasma fatty acids profile levels were previously shown to be sensitive indicators of dietary composition and ruminal biohydrogenation^22–26^. From our results, except for plasma alpha-linolenic acid concentration, the changes on plasma fatty acids concentration among ADG groups were not the same fatty acid changes as in rumen fluid. This lack of relationship between plasma and rumen fluid concentrations might partially reflect the differences between sampling time (26 d) of rumen fluid and plasma. Fatty acids in plasma are transported mainly as cholesterol esters and phospholipids within high-density lipoproteins, while triglycerides and non-esterified fatty acids typically represent less than 3% of total lipids^27^. Thus, plasma fatty acid concentrations among ADG groups might reflect non-dietary sources such as adipose and liver metabolism. In our study, the plasma concentration of 4 long-chain fatty acids (stearic acid, alpha-linolenic acid, arachidonic acid and docosahexaenoic acid) was down-regulated in the greatest-ADG group. The combination of plasma concentrations of alpha-linoleic acid and arachidonic acid to docashexaenoic acid ratio as markers of ADG in steers had a similar ROC ability (AUC = 0.86) as arachidonic acid as a single biomarker (AUC = 0.84). Taking in consideration the convenience and accessibility of blood as a testing biofluid, plasma fatty acid profile has the potential, with sufficient sensitivity and specificity, to distinguish differences in ADG.

Based on the bovine ruminal fluid metabolomics database, half of the rumen fluid metabolome (mainly longer-chain organic acid, sphingolipids, biogenic amines, and cholesterol esters) are bovine origin. The other half of the rumen fluid metabolome, which includes amino acids, fatty acids and phosphocholines is of both microbial and bovine origin^10^. In this study, steers from both treatment groups had similar DMI and received the same diet. Thus, differences in rumen fluid metabolome profile among ADG might be related to microbial fermentation and the efficient absorption of volatile fatty acids across rumen epithelia. We found higher levels of lactic acid in ruminal fluid in steers with least ADG. The increased accumulation of lactic acid might reflect an imbalance between microbial production/utilization and ruminal absorption of organic acids which has been well characterized in high grain diets^28^. For instance, changes in the diet (increase grain/decrease fiber) reduces the ruminal biohydrogenation of polyunsaturated fatty acids (PUFAs), altered the concentration of intermediate fatty acids^29^, and decreased the levels of ruminal histidine and tyrosine^6^. Additionally, most of the PUFAs have toxics effects on the ruminal microflora, suppressing, particularly, cellulolytic bacteria and fungi^30^. Indeed, inefficient dairy cows’ microbiome was charcterized by lower *Megasphaera spp*., which produce butyrate and propinate from lactate source^8^. However, in our study, ruminal’ butyrate, propionate and acetate did not differ between ADG groups. Thus, higher levels of ruminal linoleic acid and alpha-linoleic acid might negatively affect the ruminal digestibility in the least efficient steers.

Based on our analysis of the integrated pathway analysis, increased concentration of ruminal linoleic and alpha-linolenic, and decreased biosynthesis of aromatic amino acids, in less efficient steers suggest that lactic acid associated with ruminal ecosystem might affect the efficiency of overall animal production.

In summary, this study gave a new comprehensive insight into biochemical mechanisms that are associated with ADG classification. We characterized, for the first time, metabolite changes in the rumen using a distinct ruminal profiling method, identifying 33 metaboliotes associated with differences in ADG. However the causal mechanisms associated with ADG have not been determined. Linoleic and alpha-linolenic metabolism and biosynthesis of aromatic amino acid, were the most altered functional pathways associated with ADG. The combination of ruminal pentadecanoic acid, palmitic acid, linoleic acid and alpha-linolenic acid, and the combination of plasma arachidonic:docahexanoic ratio and alpha-linolenic acid, had sufficient sensitive and specificity to distinguish lower and higher ADG steers in rumen and plasma respectively. Particularly, plasma fatty acid profiles as a candidate biomarker have the potential as an accessible and useful predictive tool to further understand the mechanism underpinning ADG. In addition, the “snapshot” profile of rumen fluid, which associated the levels of linoleic/alpha-linolenic acids and aromatic amino acids with lactic acid, suggest that the balance between microbial population and ruminal absorption of organic acids affect the ADG of crossbreed beef steers.

## Materials and Methods

**Figure 5:**
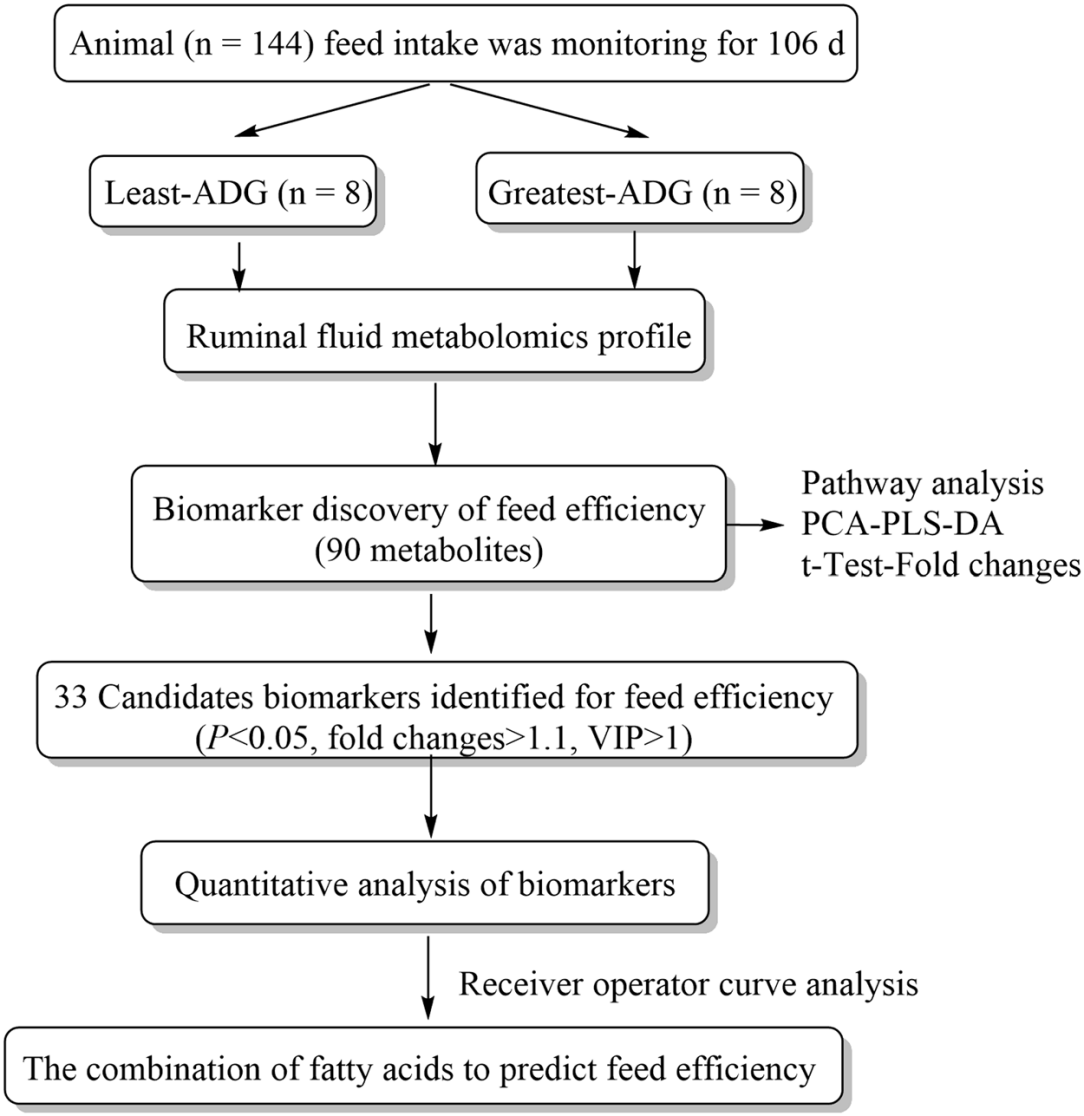
Overview of workflow utilized in the identification of biomarkers of feed efficiency on crossbreed steers.

This study was approved by the Institutional Animal Care and Use Committee (IACUC) at the U.S. Meat Animal Research Center. All experiments were performed in accordance with procedures approved by the IACUC and met the guidelines recommended in the Guide for the Care and Use of Agricultural Animals in Agricultural Research and Teaching^31^. The overall workflow utilized in the identification of biomarkers for feed efficiency is summarized in Fig. 5.

### Experimental Design

**Figure 6:**
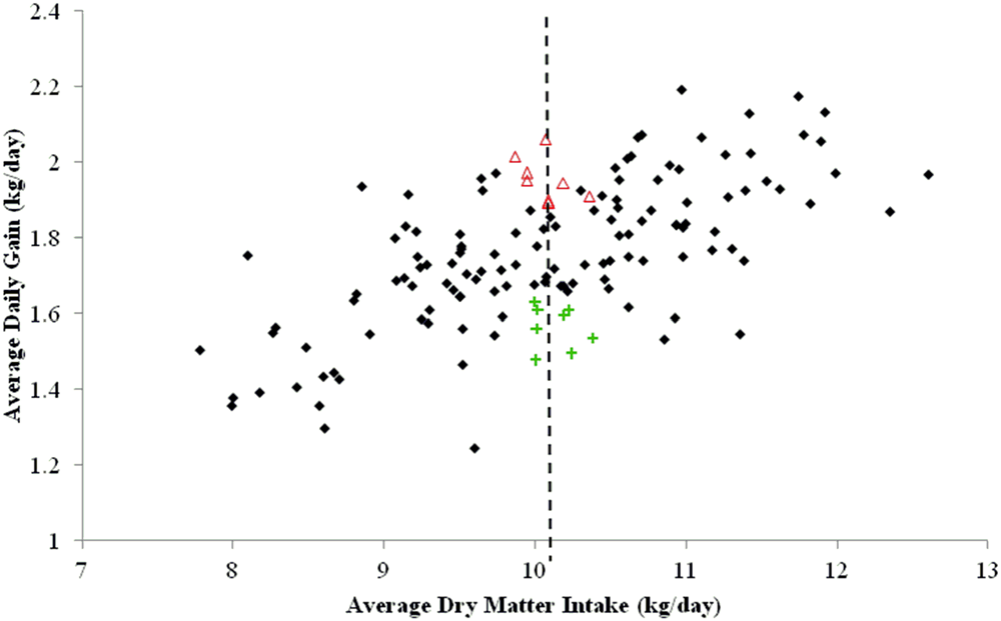
Rumen sampling selection. The two animals groups were selected by the greatest average daily gain (high-ADG; open red triangle; *n* = 8) and the least ADG steers (low-ADG; green plus; *n* = 8) with similar average dry matter intake (dash line) from the total population (black dots; *n* = 144) evaluated.

Angus-sired steers (n = 144) were used in this study. After weaning, steers received an implant (200 mg trenbolone acetate and 40 mg estradiol-17ß; Revalor-XS; Merck Animal Health, Madison, NJ, USA) and were housed in a facility with Calan Broadbent electronic headgates (American Calan, Inc., Northwood, NH) to measure individual feed intake. Steers were trained to use Calan Headgates during the adaptation period (approximately 21 d). Feed intake was measured for 105 d with cattle weighed on d 0, 1, 21, 42, 63, 84, 104, and 105 of the experiment. At the beginning of the study (0 d), mean age and weight of cattle was 310 ± 1.5 d and 403.1 ± 37.2 kg, respectively. The diet consisted, on a DM basis, of 8% chopped alfalfa hay, 20% wet distillers grains with solubles, 67.75% dry-rolled corn, and 4.25% commercial vitamin and mineral supplement; the supplement contained monensin (Rumensin 80; Elanco Animal Health, Greenfield, IN) to supply approximately 300 mg/animal daily. Cattle had been adapted to the ration for at least 35 d. The ration was mixed daily in the feed truck (Roto-Mix IV 274-12B, Dodge City, KS, USA; scale readability ±0.09 kg) according to the quantity calculated for each steer, and steers were fed once daily. The ration was sub-sampled daily, and combined to form a weekly sample to determine feed DM. Orts were measured once a week. Total DMI was the sum of total DM fed minus total orts. Initial and final BW were calculated by regressing a quadratic equation of BW on the day of study. The ADG was calculated by subtracting initial BW from final BW and dividing by days on experiment. Dry matter intake was calculated as the dry matter offered minus the orts dry matter and dividing by days on experiment. After the end of intake study, steers received the same ration *ad libitum* and remained in the same pen until slaughter (5–8 d). Blood was collected on d 84 of the feeding study via jugular venipucture into tubes containing EDTA and immediately placed on ice. Samples were centrifuged at 3,000 × g for 25 min at 4 °C and plasma was stored at −80 °C. Based on the DMI and ADG data, steers with the greatest (*n* = 8) and least (*n* = 8) ADG whose DMI was within 0.32 SD of the mean were selected for the rumen fluid study (Fig. 6). Beginning 5 d after the feeding period, 4 steers were slaughtered a day (2 steers of each treatment) for 4 consecutive days at the same time of day. Before slaughter, steers had ad libitum access to feed and water. Immediately after slaughter, the rumen of each steer was cut open and the overall rumen fluid was strained through 4 layers of cheesecloth. A 2-mL aliquot of rumen fluid was individually collected in a microcentrifuge tube and stored at −80 °C.

### Non-targeted metabolomics analysis

To optimize the sample profiling for polar and non-polar metabolites, a two-step manual solvent extraction for rumen fluid was adapted^32^. All the solvents used for extraction and chromatogram mobile phase were UPLC-MS Optima Grade (Fisher Chemical Ltd., Waltham, MA). Duplicate samples of rumen fluid were extracted for each steer. After samples were thawed on ice and vortexed, 50 mg was weighed and diluted with 1 mL of methanol/water (1:1 vol/vol), vortexed, and centrifuged at 16,000 × g for 10 min at 4 °C. The supernatant and solid precipitate were separated in different vials for aqueous (supernatant) and organic (precipitate) extraction, respectively. For the aqueous extraction, the supernatant was transferred to a new vial, dried under a nitrogen stream, and re-suspended in 500 µL methanol/water (1:1 vol/vol). For the organic extraction, the solid precipitate was dissolved in 1 mL dichlormethane/methanol (3:1 vol/vol), centrifuged (16,000 × g, 10 min at 4 °C), dried under a nitrogen stream and re-suspended in 500 µL in methanol/water (1:1 vol/vol). The UPLC/MS analysis was carried out using a Waters ACQUITY ultra-performance liquid-chromatography (UPLC) system (Waters Corp., Milford, MA) equipped with an autosampler and coupled with a hybrid triple quadrupole-time of-flight mass spectrometry (XEVO-G2-S-qTOF; Waters Corp.). Instrument calibration was performed before running the samples using 0.5 n*M* of sodium formate solution. The injection of the ruminal fluid extraction volume was 10 µL and the sample temperature was 4 °C. To obtain information regarding system suitability and stability, quality control (QC) samples were injected at regular intervals throughout the analytical run. Quality control samples were prepared by mixing rumen fluid extraction aliquots (10 µL), producing separate QC samples for aqueous and organic extracts.

Liquid chromatography for the aqueous extraction was carried out using an Acquity UPLC BEH HILIC column (2.1 × 100 mm × 1.7 µm; Waters Corp.). The separation was performed at 0.5 mL of flow rate and 30 °C of column temperature. The mobile phase A was 0.1% formic acid with 10 m*M* ammonium acetate at pH 8.0 in water and mobile phase B was 0.1% formic acid in acetonitrile:water (95:5 vol/vol) with 10 m*M* ammonium acetate at pH 8.0. The gradient of mobile phase B:A was 99:1 to 0.1:99.9 in 5 min followed by 2 min of re-equilibration at initial conditions.

Liquid chromatography for the organic extraction was carried out using an Acquity UPLC BEH (Ethylene Bridged Hybrid Technology; Waters Corp.) C18 (2.1 × 50 mm × 1.7 µm) column performed at 0.4 mL min of flow rate and 40 °C of column temperature. The mobile phase A was 0.1% formic acid in water and mobile phase B 0.1% formic acid in acetonitrile. The gradient of mobile phase A:B was 85:15:0% for 2 min, ramp to 50:50 at 5 min, and ramp to 99.9:0.01% at 18 min, to reach the initial state at min 19 followed by 3 min of re-equilibration.

Mass spectrometry was performed in both positive and negative modes. The capillary voltage was 3.2 kV and 2.4 kV for positive and negative mode, respectively. The system parameters were set as follows: source of temperature 120 °C, desolvation temperature 350 °C, cone gas flow (nitrogen) 25 L/h and desolvation gas flow (nitrogen) 900 L/h. Data were collected in a centroid mode using the lockspray to ensure accuracy and reproducibility. Leucine enkephalin was used as lock-mass in a 2 ng/mL concentration solution. The lock spray frequency was set at 15 s, and the lock mass data were average over 15 scans for correction. The scan mass range was from 50–1200 m/z using an extended dynamic range. The MS/MS analysis was carried out by ramping the collision energy from 10 to 50 V using argon as a collision gas.

### Data and statistical analysis (non-targeted-metabolomics)

Raw data were analyzed using Progenesis QI v1.0 software (Waters Corp.). The data was aligned and normalized using total ion intensity. The ruminal compounds were identified by comparison with online Bovine Metabolome Database (http://www.cowmetdb.ca/ *)* using exact m/z values and retention times. The *t*-test and principal components analysis (PCA) were conducted to identify and visualize differences from least-ADG vs. greatest-ADG steers; data were filtered using a significance level of *P* < 0.10 (Table 1). PROC MULTTEST procedure of SAS 9.3 (SAS Inst. Inc., Cary, NC) was conducted to multiplicity-adjust a collection of raw *p*-values. Data tested for normality were log transformed and standardized using Pareto scaling technique. Pathway analysis was performed using a *Bos taurus* pathway library, which integrate global pathway enrichment analysis and relative between centrality pathway topology analysis from MetaboAnalyst 3.0 software. The identification and visualization of the top altered pathway were based on KEGG (http://www.genome.jp/kegg/) database sources. The importance of a metabolite, within a given metabolic network, is calculated by its centrality measures (degree centrality and betweenness centrality), measuring the number of connections the pathway of interest has to other pathways and the latter measures the number of shortest paths going through the pathway of interest^33^. Pathway impact is calculated adding up the importance measures of each of the matched metabolites and then diving by the sum of the importance measures of all metabolites in each pathway.

To identify potentially different metabolites, univariate and multivariate analyses were also performed using MetaboAnalyst 3.0 software according to previously published recommended statistical procedure for metabolomics analysis^33–37^.

Partial least squares-discriminate analysis (PLS-DA) was conducted to identify the significant metabolites responsible for the differentiation of least-ADG and greatest-ADG steers, using a significance levels of *P* < 0.05, fold change >1.1, and variable of importance in projection (VIP > 1.0). The PLS-DA model is a type of partial least squares (PLS) regression where the dependent variable is a binary outcome (i.e., greater-ADG vs. least-ADG) and the independent variables are metabolites detected and selected based on criteria of VIP. The VIP values are produced by PLS-DA model and represent the weighted sum of squares of the PLS loading, which takes into account the amount of orthogonal variance explained by each component^35^.

Variable importance in the projection with values >1.0 suggest that the metabolite is significantly involved in the separation of groups^37^. Permutation testing (100 times) was performed to minimize the possibility that observed separation on a PLS-DA was by chance.

A receiver-operator characteristic curve (ROC) was calculated by ROCCET web service used to evaluate predictive ability of potential metabolic biomarkers^35^. Area under the curve (AUC) from ROC curve was the metric used to interpret the performance across different biomarkers models to determine the best cut off point for sensitivity and specificity. In the context of our experiment designed to discriminate between binary outcome on ADG: sensitivity is the probability of a positive result from a steer with actual true positive outcome (true positive), and specificity is the probability of a negative test result from a steer with actual negative outcome (true negative).

The identities of selected biomarkers were confirmed by MS/MS fragment ion analysis using Mass-Fragment application manager software (Water MassLynk v4.1, Waters Corp.). The MS/MS fragmentation of the candidate molecules was compared, with ChemSpider database (www.chemspider.com), by way of chemically intelligent peak-matching algorithms.

### Quantitative analysis of biomarkers

In order to validate the biomarkers for feed efficiency, metabolites selected from the biodiscovery analysis (according to *t*-test, AUCs, and pathway analysis) were quantified. The saturated/unsaturated fatty acids were quantified using isotope dilution MS methodology based on Isaac *et al*.^38^. As well, plasma fatty acids were quantified and correlated with ruminal fatty acid concentration to identify accessible markers of feed efficiency. The stock solution of linoleic acid-D_11_ (Cayman 9002193, Ann Arbor, MI), used as internal standard (IS), was prepared in chloroform: methanol (2:1, vol/vol) at the concentration of 0.5 µg/mL. The calibration curve for saturated/monounsaturated fatty acids mixture (Cayman17942) and for polyunsaturated fatty acid mixture (Cayman17941) were prepared by serial dilution (12-fold) of 10 µg/mL in isopropanol:acetonitrile:water (2:1:1, vol/vol/v). Pentadecanoic acid was not present in the fatty acid mixture; therefore, an individual calibration curve was formed by serial diluting 24 µg/ml (Sigma-Aldrich M9005, St. Louis, MO).

Rumen fluid and plasma samples were thawed on ice and 200 µL was extracted with 800 µL of chlorform:methanol (2:1, vol/vol) and 20 µL of IS (linoleic acid-D_11_). Samples were vortexed and centrifuged at 12,000 × g, for 5 min at 4 °C. The lower organic phase was collected in a new vial and evaporated under nitrogen stream. The samples were re-diluted with 500 µL of isopropanol:acetonitrile:water (2:1:1, vol/vol/vol).

Fatty acids were quantified using a UPLC- XEVO-G2-S-qTOF (Waters Corp.). The injection volume of the extraction was 10 µL and the autosample temperature was 4 °C. The separation was performed using an Acquity UPLC charged surface hybrid technology column C18 (2.1 × 100 mm × 1.7 µm; Waters) at 0.4 mL/min and 55 °C. Mobile phase A was acetonitrile:water (60:40, vol/vol) and mobile phase B was isopropanol:acetonitrile (90:10, vol/vol), and both were prepared with 10 n*M* ammonium formate and 0.1% formic acid. The elution gradient was as follows: 70% A for 1 min, 70 to 67% over 1 min, 67 to 57% over 1 min, 57 to 50% over 1 min, 50 to 30% over 4 min, 30 to 1% over 1 min and held for 2 min, returned to initial conditions in 10 s and maintained for 2 min.

The mass spectrometry was performed in negative ionization mode. The parameters were as follows: capillary voltage 1000 V, sample cone voltage 30 V, source temperature 120 °C and desolvation gas (nitrogen) gas 900 L/hr. The mass acquisition rate was set at 0.2 s. The scan mass range was from 100 to 500 m/z. The qTOF-MS/MS data were collected in centroid mode using the lock-mass.

The data acquisition was performed using MassLynk software (version 4.1, Waters Corporation, Milford, USA) and process and quantification of fatty acids was determined by TargetLynk application manager. Differences between the two groups were analyzed by an independent t-test and area under the curve (AUC) performed by MetaboAnalyst 3.0 software.

## References

[1] Freetly HC, Brown-Brandl TM (2013). Enteric methane production from beef cattle that vary in feed efficiency. J. Anim. Sci..

[2] Herd RM, Oddy VH, Richardson EC (2004). Biological basis for variation in residual feed intake in beef cattle. 1. Review of potential mechanisms. Aust. J. Exp. Agr..

[3] Myer PR (2015). Rumen microbiome from steers differing in feed efficiency. PloS one.

[4] Hernandez-Sanabria E (2012). Impact of feed efficiency and diet on adaptive variations in the bacterial community in the rumen fluid of cattle. Appl. Environ. Microbiol..

[5] Ametaj BN (2010). Metabolomics reveals unhealthy alterations in rumen metabolism with increased proportion of cereal grain in the diet of dairy cows. Metabolomics.

[6] Saleem F (2012). A metabolomics approach to uncover the effects of grain diets on rumen health in dairy cows. J. Dairy Sci..

[7] Zhao S (2014). Metabolomics analysis reveals large effect of roughage types on rumen microbial metabolic profile in dairy cows. Lett. Appl. Microbiol..

[8] Shabat SKB (2016). Specific microbiome-dependent mechanisms underlie the energy harvest efficiency of ruminants. ISME J.

[9] Berry DP (2011). The integration of ‘omic’ disciplines and systems biology in cattle breeding. Animal.

[10] Saleem F (2013). The bovine ruminal fluid metabolome. Metabolomics.

[11] Chen H (2015). The metabolome profiling and pathway analysis in metabolic healthy and abnormal obesity. International journal of obesity.

[12] Cao, J. *et al*. Effects of MeJA on Arabidopsis metabolome under endogenous JA deficiency. *Scientific Reports* **6** (2016). 10.1038/srep37674 PMC5121592 27883040

[13] Shrinet, J. *et al*. Serum metabolomics analysis of patients with chikungunya and dengue mono/co-infections reveals distinct metabolite signatures in the three disease conditions. *Scientific Reports* **6** (2016). 10.1038/srep36833 PMC5109290 27845374

[14] Jenkins TC (1993). Lipid metabolism in the rumen. J. Dairy Sci.

[15] Harfoot, C. G. & Hazlewood, G. P. In *The Rumen Microbial* Ecosystem (eds Hobson, P. N. & Stewart, C. S.) 382–426 (Springer Netherlands, 1997).

[16] Buccioni A (2012). Lipid metabolism in the rumen: New insights on lipolysis and biohydrogenation with an emphasis on the role of endogenous plant factors. Anim. Feed Sci.Tech..

[17] Jenkins B, West JA, Koulman A (2015). A review of odd-chain fatty acid metabolism and the role of pentadecanoic acid (C15: 0) and heptadecanoic acid (C17: 0) in health and disease. Molecules.

[18] Jami E, White BA, Mizrahi I (2014). Potential role of the bovine rumen microbiome in modulating milk composition and feed efficiency. PLoS One.

[19] Kim EJ (2008). Fish oil increases the duodenal flow of long chain polyunsaturated fatty acids and trans-11 18:1 and decreases 18:0 in steers via changes in the rumen bacterial community. J. Nutr..

[20] Huws SA (2009). Rumen protozoa are rich in polyunsaturated fatty acids due to the ingestion of chloroplasts. FEMS Microbiol. Ecol.

[21] Shingfield KJ (2012). Dietary fish oil supplements modify ruminal biohydrogenation, alter the flow of fatty acids at the omasum, and induce changes in the ruminal Butyrivibrio population in lactating cows. J. Nutr..

[22] Loor J, Herbein J, Jenkins T (2002). Nutrient digestion, biohydrogenation, and fatty acid profiles in blood plasma and milk fat from lactating Holstein cows fed canola oil or canolamide. Anim. Feed Sci. Tech..

[23] Vasta V (2009). Metabolic fate of fatty acids involved in ruminal biohydrogenation in sheep fed concentrate or herbage with or without tannins. J. Anim. Sci..

[24] Kay JK (2005). A comparison between feeding systems (pasture and TMR) and the effect of vitamin E supplementation on plasma and milk fatty acid profiles in dairy cows. J. Dairy Res..

[25] Sinclair L (2007). Biohydrogenation of n-3 polyunsaturated fatty acids in the rumen and their effects on microbial metabolism and plasma fatty acid concentrations in sheep. Anim. Sci..

[26] Alvarado-Gilis C (2015). Effects of flaxseed encapsulation on biohydrogenation of polyunsaturated fatty acids by ruminal microorganisms: feedlot performance, carcass quality, and tissue fatty acid composition. J. Anim. Sci.

[27] Moore JH, Christie WW (1979). Lipid metabolism in the mammary gland of ruminant animals. Prog. Lipid Res..

[28] Nagaraja TG, Titgemeyer EC (2007). Ruminal acidosis in beef cattle: the current microbiological and nutritional outlook. J. Dairy Sci..

[29] Loor JJ (2004). Biohydrogenation, duodenal flow, and intestinal digestibility of trans fatty acids and conjugated linoleic acids in response to dietary forage:concentrate ratio and linseed oil in dairy cows. J. Dairy Sci..

[30] Maia MRG, Chaudhary LC, Figueres L, Wallace RJ (2007). Metabolism of polyunsaturated fatty acids and their toxicity to the microflora of the rumen. Antonie van Leeuwenhoek.

[31] FASS. 2010. Guide for the Care and Use of Agricultural Animals in Agricultural. Research and Teaching. 3rd ed. Consortium for Developing a Guide for the Care and Use of Agricultural Animals in Agricultural Research and Teaching, Champaign, IL.

[32] Want EJ (2013). Global metabolic profiling of animal and human tissues via UPLC-MS. Nat. Protocols.

[33] Xia J, Wishart DS (2011). Web-based inference of biological patterns, functions and pathways from metabolomic data using MetaboAnalyst. Nat. Protoc..

[34] Xia J (2012). MetaboAnalyst 2.0–a comprehensive server for metabolomic data analysis. Nucleic Acids Res.

[35] Xia J, Broadhurst DI, Wilson M, Wishart DS (2013). Translational biomarker discovery in clinical metabolomics: an introductory tutorial. Metabolomics.

[36] Xia J, Sinelnikov IV, Han B, Wishart DS (2015). MetaboAnalyst 3.0–making metabolomics more meaningful. Nucleic Acids Res..

[37] Xia J, Psychogios N, Young N, Wishart DS (2009). MetaboAnalyst: a web server for metabolomic data analysis and interpretation. Nucleic Acids Res.

[38] Isaac, G., McDonald, S. & Astarita, G. Lipid separation using UPLC with charged surface hybrid technology. *Waters App note*. http://www.waters.com/webassets/cms/library/docs/720004107en.pdf (Date of access: 02/12/2016) (2011).

